# Reverse left ventricular remodeling after aortic valve replacement for aortic stenosis: a systematic review and meta-analysis

**DOI:** 10.3389/fcvm.2024.1407566

**Published:** 2024-07-04

**Authors:** F. Sousa Nunes, C. Amaral Marques, A. Isabel Pinho, B. Sousa-Pinto, A. Beco, J. Ricardo Silva, F. Saraiva, F. Macedo, A. Leite-Moreira, C. Sousa

**Affiliations:** ^1^ Cardiovascular R&D Centre—UnIC@RISE, Department of Surgery and Physiology, Faculty of Medicine of the University of Porto, Porto, Portugal; ^2^ Department of Cardiology, Local Health Unit of Gaia and Espinho, Vila Nova de Gaia, Portugal; ^3^ Department of Cardiology, Local Health Unit of Sao Joao, Porto, Portugal; ^4^ MEDCIDS—Department of Community Medicine, Information and Health Decision Sciences, Faculty of Medicine, University of Porto, Porto, Portugal; ^5^ CINTESIS@RISE—Health Research Network, MEDCIDS, Faculty of Medicine, University of Porto, Porto, Portugal

**Keywords:** aortic stenosis, transcatheter aortic valve implantation (TAVI), surgical aortic valve replacement (SAVR), reverse left ventricle remodeling, echocardiography

## Abstract

Reverse left ventricular (LV) remodeling after aortic valve replacement (AVR), in patients with aortic stenosis, is well-documented as an important prognostic factor. With this systematic review and meta-analysis, we aimed to characterize the response of the unloaded LV after AVR. We searched on MEDLINE/PubMed and Web of Science for studies reporting echocardiographic findings before and at least 1 month after AVR for the treatment of aortic stenosis. In total, 1,836 studies were identified and 1,098 were screened for inclusion. The main factors of interest were structural and dynamic measures of the LV and aortic valve. We performed a random-effects meta-analysis to compute standardized mean differences (SMD) between follow-up and baseline values for each outcome. Twenty-seven studies met the eligibility criteria, yielding 11,751 patients. AVR resulted in reduced mean aortic gradient (SMD: −38.23 mmHg, 95% CI: −39.88 to −36.58, $I^{2}=92\%$), LV mass (SMD: −37.24 g, 95% CI: −49.31 to −25.18, $I^{2}=96\%$), end-diastolic LV diameter (SMD: −1.78 mm, 95% CI: −2.80 to −0.76, $I^{2}=96\%$), end-diastolic LV volume (SMD: −1.6 ml, 95% CI: −6.68 to 3.51, $I^{2}=91\%$), increased effective aortic valve area (SMD: 1.10 cm^2^, 95% CI: 1.01 to 1.20, $I^{2}=98\%$), and LV ejection fraction (SMD: 2.35%, 95% CI: 1.31 to 3.40%, $I^{2}=94.1\%$). Our results characterize the extent to which reverse remodeling is expected to occur after AVR. Notably, in our study, reverse remodeling was documented as soon as 1 month after AVR.

## 1 Introduction

Aortic stenosis (AS) is the most common acquired valvopathy in the Western world (1). Its incidence increases with age, and its prevalence is expected to rise in the future (2).

AS is not an isolated valve disease but a more complex and broad pathology involving the myocardium. AS progression is associated with left ventricular (LV) remodeling, which is the myocardial response to increased afterload (2). Initially, LV remodeling is a compensatory response to a persistent obstacle to systolic ejection. The sustained increased pressure and hemodynamic load lead to the classical development of LV hypertrophy. This initial adaptation allows for a reduction in wall stress and maintenance of cardiac output. After this stage, persistent obstruction leads to maladaptive LV remodeling, causing gradual deterioration of diastolic and systolic functions (1). Clinically, this process can translate into various symptoms, including death due to heart failure or arrhythmic events (2). In other words, maladaptive LV response negatively impacts the prognosis of AS patients regarding survival and cardiovascular events (3).

The only effective treatment for severe AS is aortic valve replacement (AVR), which can be performed either surgically (SAVR) or percutaneously via transcatheter AV implantation (TAVI). AVR aims to eliminate the LV obstruction and ultimately revert this inadequate LV response (2). After AVR, the extension of the achieved reverse LV remodeling is a major determinant of symptoms and outcomes (2). Its prognostic importance has been reported in several randomized trials (2, 4, 5). Transthoracic echocardiography (TTE) is the gold standard method to characterize AS severity, LV remodeling, and LV reverse remodeling after AVR. These LV adaptations comprise several changes in echocardiographic parameters, such as LV mass, cavity dimensions and volumes, wall thicknesses, and left ventricular ejection fraction (LVEF) (1). Unfortunately, data to predict LV response after AVR are lacking.

In this systematic review and meta-analysis, we aim to assess the extent of left ventricular remodeling at pre-determined time points post-procedure in patients with aortic stenosis who underwent AVR. The measured variables of interest included effective aortic valve area (AVA), mean aortic gradient (MAG), left ventricular mass (LVM), LVEF, and end-diastolic left ventricular diameter (EDLVD) and volume (EDLVV).

## 2 Methods

### 2.1 Eligibility and search strategy

This systematic review and meta-analysis was conducted according to the PRISMA (Preferred Reporting Items for Systematic Reviews and Meta-Analyses) statement (6).

The literature search was conducted on 15 March 2022 in two electronic databases: MEDLINE (through PubMed) and Web of Science. The search was conducted with no restrictions on language or year of publication. Full details of the search are presented in Table 1.

**Table 1:** Keywords used to perform the query in the two databases used in this study (date of search: 15 March 2022).

ISI Web of Knowledge	(TS = (“ventricular mass”) OR TS = (“LV mass”) OR TS = (“septum thickness”) OR TS = (“posterior wall thickness”) OR TS = (“mass regression”) OR TS = (“end diastolic diameter”) OR TS = (“end systolic diameter”) OR TS = (“end diastolic volume”) OR TS = (“end systolic volume”) OR TS = (“remodeling”) OR TS = (“remodelling”) OR TS = (“LVEDD”) OR TS = (“LVESD”)
	AND
	TS = (“TAVI”) OR TS = (“TAVR”) OR TS = (“aortic valve replacement”) OR TS = (“aortic valve implantation”) OR TS = (“AVR”) OR TS = (“prosthesis implantation”)
	AND
	TS = (“patients”) OR TS = (“patient”) OR TS = (“subjects”))
	NOT
	(TI = (“aortic insufficiency”) OR TI = (“aortic regurgitation”) OR TS = (“magnetic resonance”) OR TS = (“computed tomography”)
	OR
	DT = (Editorial Material) OR DT = (Review))
MEDLINE/PubMed	((“TAVI”[Title/Abstract] OR “TAVR”[Title/Abstract] OR “aortic valve replacement”[Title/Abstract] OR “aortic valve implantation”[Title/Abstract] OR “AVR”[Title/Abstract] OR “prosthesis implantation”[Title/Abstract])
	AND
	(“ventricular mass”[Title/Abstract] OR “LV mass”[Title/Abstract] OR “septum thickness”[Title/Abstract] OR “posterior wall thickness”[Title/Abstract] OR “mass regression”[Title/Abstract] OR “end diastolic diameter”[Title/Abstract] OR “end systolic diameter”[Title/Abstract] OR “end diastolic volume”[Title/Abstract] OR “end systolic volume”[Title/Abstract] OR “remodeling”[Title/Abstract] OR “remodelling”[Title/Abstract] OR “LVEDD”[Title/Abstract] OR “LVESD”[Title/Abstract])
	AND
	(“patients”[Title/Abstract] OR “patient”[Title/Abstract] OR “subjects”[Title/Abstract]))
	NOT
	(“editorial”[Publication Type] OR “review”[Publication Type] OR “systematic review”[Publication Type] OR “Case Reports”[Publication Type] OR “aortic insufficiency”[Title] OR “aortic regurgitation”[Title] OR “magnetic resonance”[Title/Abstract] OR “computed tomography”[Title/Abstract])

Studies were included if they reported echocardiographic findings before and at least 1 month after SAVR or TAVI for the treatment of AS. This time interval was chosen to allow acute changes after the procedure to resolve and for reverse remodeling to occur (7). Furthermore, patient evaluation had to be performed at pre-determined time points post-procedure, i.e., at either 1, 3, 6, or 12 months.

Studies also needed to report at least one outcome variable of interest for the measurement of the left ventricle reverse remodeling to be included, namely, left ventricular dimensions or ejection fraction.

We excluded all non-human studies, case–control studies, case reports, and reviews. Studies without a predefined follow-up period and with fewer than 100 patients were also excluded.

### 2.2 Study selection, data collection process, and study outcomes

Two investigators (FSN and CAM) independently reviewed each study by title and abstract and then by full-text reading. Discordant decisions were managed by consensus. Authors of primary studies were contacted for clarification if relevant data were missing. For each primary study, two investigators (FSN and CAM) independently performed data extraction. We extracted the following information: study design (clinical setting, duration of follow-up, and number of patients included), Baseline characteristics of the population (Table 2) [eligibility criteria; age; gender; New York Heart Association (NYHA) class; body surface area (BSA); and frequency of hypertension, diabetes mellitus (DM), coronary heart disease, and other comorbidities], intervention (details on SAVR or TAVI procedures), and outcome data of interest. The latter included effective AVA, MAG, EDLVD, EDLVV, LVM, and LVEF.

**Table 2:** Baseline characteristics of included studies.

	Cohort (if applicable)	Female sex (%)	Age^a^ (years)	NYHA III/IV (%)	HTN (%)	Diabetes (%)	CAD (%)	BSA (m^2^)^b^	Initial LVEF >50% (Y/N)	Follow-up (months)	Evaluation dates	Valve
Campos et al. (8)		47.6	70.9 ± 7.5	60.9	NR	NR	22.4	NR	NR	6	1993–2004	Biological
Gegenava et al. (9)		50	80 ± 7	57	76	26	60	NR	NR	12	NR	Biological
Ngo et al. (10)		43.3	79 ± 5	50.4	72	17.6	4.2	1.9 (0.2)	NR	3 and 12	2009–2014	Biological
Gelsomino et al. (11)		50.4	71.3 ± 6.4	93.6	NR	NR	30.4	1.7 (0.1)	NR	6 and 12	1993–2000	Biological
Vizzardi et al. (12)		48	83 ± 7	55	68	47	25	1.7 (0.17)	NR	6	NR	Biological
Pibarot et al. (13)	TAVI	32.5	73.3 ± 5.8	31.3	85	31.3	27.7	2 (0.2)	NR	1 and 12	2016–2017	Biological
	SAVR	28.9	73.6 ± 6.1	23.8	85.9	30.2	28	2 (0.2)	NR	1 and 12	2016–2017	Biological
Izumi et al. (14)		53	70 ± 9	19	43	17	11	1.52 (0.17)	NR	12	2000–2006	Both
Harrington et al. (15)		45.6	81 ± 9	NR	91	28	64	1.9 (0.3)	NR	12	2011–2017	Biological
Merdler et al. (16)		54.5	82 ± 6.1	85.6	86.1	37.7	50.1	NR	NR	12	2009–2018	Biological
Martinovic et al. (17)		57	72.8 ± 3	81	65	22	45	NR	NR	12	1996–2004	Biological
Al-Rashid et al. (18)		52.3	82 ± 4	91.3	85.3	31.3	NR	NR	NR	3	2016–2017	Biological
Thomson (19)		46.5	74 ± 6	NR	NR	NR	NR	NR	NR	6	1992–1997	Biological
Ewe et al. (20)		60.7	81.1 ± 6.2	83	75.5	17	NR	1.73 (0.18)	NR	6	NR	Biological
Douglas et al. (21)		55.2	83.2 ± 8.9	NR	86.6	31.5	69.9	NR	NR	1	2007–2010	Biological
Ledwoch et al. (22)		39	79 ± 8	63	90	22	68	NR	NR	12	2015–2020	Biological
Al-Hijji et al. (23)		47.8	82.5 ± 7.7	86.1	88.7	38.3	NR	NR	NR	1	2012–2016	Biological
Weber et al. (24)		37	NR	54	81	26	45	NR	NR	3	2015–2016	Biological
Fuster et al. (25)		36.2	63 ± 9	70	39.5	16.2	NR	1.7 (0.2)	NR	1	1994–2001	Both
Theron et al. (26)		31.3	76.8 ± 6.2	35.3	99.3	27.3	NR	NR	NR	1 and 12	2012–2015	Biological
Chau et al. (27)		53	84 ± 7	NR	94	36	77	1.81 (0.24)	NR	1 and 12	2007–2020	Biological
Ochiai et al. (28)	RAS	70.1	84.2 ± 5	46.6	83.8	27.8	41.5	1.44 (0.16)	NR	6	2013–2016	Biological
	No RAS	75.7	84.8 ± 5	54.5	61.4	24.3	33.9	1.39 (0.17)	NR	6	2013–2016	Biological
Little et al. (29)	TAVI	47	83.2 ± 7.1	85.7	NR	NR	75.3	1.8 (0.2)	NR	12	2010–2014	Biological
	SAVR	48.2	83.3 ± 6.4	87	NR	NR	75.6	1.9 (0.2)	NR	12	2010–2014	Biological
Ninomiya et al. (30)		58	83.2 ± 5	NR	NR	43	40	1.5 (0.2)	NR	3	2013–2018	Biological
Iliopoulos et al. (31)		50	75.8 ± 5.1	25.7	93	35.9	64.8	1.7 (0.2)	NR	3, 6 and 12	2006–2010	Biological
Beholz et al. (32)		55	76.5 ± 6.4	63	73	24	NR	NR	NR	1 and 12	2004–2006	Biological
Fischlein et al. (33)		64.4	78.3 ± 5.6	63.7	83.7	29	NR	1.8 (0.2)	NR	12	2010–2013	Biological
Medvedofsky et al. (34)		100	83 ± 8	84	94	36	NR	NR	NR	12	2007–2014	Biological

BSA, body surface area; CAD, coronary artery disease; HTN, hypertension; LVEF, left ventricle ejection fraction; NYHA, New York Heart Association (NYHA) Classification; NR, not reported; RAS, renin angiotensin system therapy; SAVR, surgical aortic valve replacement; TAVI, transcatheter aortic valve implantation.

^a^mean  ±  standard deviation (SD).

^b^median (interquartile range).

### 2.3 Risk of bias assessment

We used the Study Quality Assessment Tool for Observational Cohort Studies from the National Institutes of Health to categorize several domains for all the eligible studies. The overall risk of bias was independently assigned to each study by two investigators (FSN, CAM) and classified into “good,” “fair,” and “poor”, as detailed in Table 3.

**Table 3:** Risk of bias assessment of the included studies.

Criteria	Campos et al. (8)	Gegenava et al. (9)	Ngo et al. (10)	Gelsomino et al. (11)	Vizzardi et al. (12)	Pibarot et al. (13)	Izumi et al. (14)	Harrington et al. (15)	Merdler et al. (16)	Martinovic et al. (17)	Al-Rashid et al. (18)	Thomson (19)	Ewe et al. (20)	Douglas et al. (21)	Ledwoch et al. (22)	Al-Hijji et al. (23)
1. Was the research question or objective in this paper clearly stated?	Yes	Yes	Yes	Yes	Yes	Yes	Yes	Yes	Yes	Yes	Yes	Yes	Yes	Yes	Yes	Yes
2. Was the study population clearly specified and defined?	Yes	No	Yes	Yes	Yes	Yes	Yes	Yes	Yes	Yes	Yes	No	Yes	Yes	Yes	Yes
3. Was the participation rate of eligible persons at least 50%?	NA	NA	NR	NA	NA	NR	NA	NA	NA	NA	NA	NA	NA	NR	NA	NA
4. Were all the subjects selected or recruited from the same or similar populations (including the same time period)? Were inclusion and exclusion criteria for being in the study prespecified and applied uniformly to all participants?	Yes	NR	Yes	Yes	Yes	Yes	Yes	Yes	Yes	Yes	Yes	Yes	Yes	Yes	Yes	Yes
5. Was a sample size justification, power description, or variance and effect estimates provided?	No	No	No	No	No	No	No	No	No	No	No	No	No	NR	No	No
6. For the analyses in this paper, were the exposure(s) of interest measured prior to the outcome(s) being measured?	Yes	Yes	Yes	Yes	Yes	Yes	Yes	Yes	Yes	Yes	Yes	Yes	Yes	Yes	Yes	Yes
7. Was the timeframe sufficient so that one could reasonably expect to see an association between exposure and outcome if it existed?	Yes	Yes	Yes	Yes	Yes	Yes	Yes	Yes	Yes	Yes	Yes	Yes	Yes	Yes	Yes	Yes
8. For exposures that can vary in amount or level, did the study examine different levels of the exposure as related to the outcome (e.g., categories of exposure, or exposure measured as continuous variable)?	Yes	Yes	Yes	Yes	No	Yes	No	No	No	Yes	Yes	No	Yes	Yes	Yes	Yes
9. Were the exposure measures (independent variables) clearly defined, valid, reliable, and implemented consistently across all study participants?	Yes	No	Yes	Yes	Yes	Yes	Yes	Yes	Yes	No	Yes	Yes	Yes	Yes	Yes	Yes
10. Was the exposure(s) assessed more than once over time?	Yes	Yes	Yes	Yes	Yes	Yes	Yes	Yes	Yes	Yes	Yes	Yes	Yes	Yes	Yes	Yes
11. Were the outcome measures (dependent variables) clearly defined, valid, reliable, and implemented consistently across all study participants?	Yes	Yes	Yes	Yes	Yes	Yes	Yes	Yes	Yes	Yes	Yes	Yes	Yes	Yes	Yes	Yes
12. Were the outcome assessors blinded to the exposure status of participants?	No	No	No	No	No	No	No	No	No	No	No	No	No	No	No	No
13. Was loss to follow-up after baseline 20% or less?	Yes	Yes	Yes	Yes	Yes	Yes	Yes	No	NR	NR	Yes	NR	Yes	NR	Yes	NR
14. Were key potential confounding variables measured and adjusted statistically for their impact on the relationship between exposure(s) and outcome(s)?	No	No	No	No	No	No	No	No	No	No	No	No	No	No	No	No
Overall risk of bias	Good	Good	Good	Good	Good	Good	Good	Good	Good	Good	Good	Good	Good	Good	Good	Good

Criteria	Weber et al. (24)	Fuster et al. (25)	Theron et al. (26)	Chau et al. (27)	Ochiai et al. (28)	Little et al. (29)	Ninomiya et al. (30)	Iliopoulos et al. (31)	Beholz et al. (32)	Fischlein et al. (33)	Medvedofsky et al. (34)
1. Was the research question or objective in this paper clearly stated?	Yes	Yes	Yes	Yes	Yes	Yes	Yes	Yes	Yes	Yes	Yes
2. Was the study population clearly specified and defined?	Yes	Yes	Yes	Yes	Yes	Yes	Yes	Yes	Yes	Yes	Yes
3. Was the participation rate of eligible persons at least 50%?	NA	NA	NA	NR	NA	NA	NA	NA	NA	NA	No
4. Were all the subjects selected or recruited from the same or similar populations (including the same time period)? Were inclusion and exclusion criteria for being in the study prespecified and applied uniformly to all participants?	Yes	Yes	Yes	Yes	Yes	Yes	Yes	Yes	Yes	Yes	Yes
5. Was a sample size justification, power description, or variance and effect estimates provided?	No	No	No	NR	NR	NR	No	No	No	No	No
6. For the analyses in this paper, were the exposure(s) of interest measured prior to the outcome(s) being measured?	Yes	Yes	Yes	Yes	Yes	Yes	Yes	Yes	Yes	Yes	Yes
7. Was the timeframe sufficient so that one could reasonably expect to see an association between exposure and outcome if it existed?	Yes	Yes	Yes	Yes	Yes	Yes	Yes	Yes	Yes	Yes	Yes
8. For exposures that can vary in amount or level, did the study examine different levels of the exposure as related to the outcome (e.g., categories of exposure, or exposure measured as continuous variable)?	Yes	Yes	Yes	Yes	Yes	Yes	Yes	Yes	Yes	Yes	Yes
9. Were the exposure measures (independent variables) clearly defined, valid, reliable, and implemented consistently across all study participants?	Yes	Yes	Yes	Yes	Yes	Yes	Yes	Yes	Yes	Yes	Yes
10. Was the exposure(s) assessed more than once over time?	Yes	Yes	Yes	Yes	Yes	Yes	Yes	Yes	Yes	Yes	Yes
11. Were the outcome measures (dependent variables) clearly defined, valid, reliable, and implemented consistently across all study participants?	Yes	Yes	Yes	Yes	Yes	Yes	Yes	Yes	Yes	Yes	Yes
12. Were the outcome assessors blinded to the exposure status of participants?	No	No	No	No	No	No	No	No	No	No	No
13. Was loss to follow-up after baseline 20% or less?	Yes	No	Yes	NR	Yes	NR	Yes	Yes	Yes	Yes	Yes
14. Were key potential confounding variables measured and adjusted statistically for their impact on the relationship between exposure(s) and outcome(s)?	No	No	No	No	No	No	No	No	No	No	No
Overall risk of bias	Good	Good	Good	Good	Good	Good	Good	Good	Good	Good	Good

NA, not applicable; NR, not reported.

### 2.4 Statistical analysis

We performed a random-effects meta-analysis using the restricted maximum likelihood approach to compute pooled mean differences (MD) or standardized mean differences (SMD) between post-follow-up and baseline values for each outcome. Heterogeneity was assessed by the Cochran Q statistic p-value and the $I^{2}$ statistic: a p-value <0.10 and an $I^{2}$ >50% were considered to represent substantial heterogeneity. Sources of heterogeneity were explored using univariable meta-regression models, with tested covariates including the publication year, mean age of the participants, percentage of females, average BSA, percentage of patients in NYHA classes III/IV, and percentage of patients with other comorbidities such as hypertension, diabetes, and coronary heart disease. In addition, we performed subgroup analyses for the follow-up period and the initial LVEF (classes were categorized into two groups: lower than 50% and higher than 50%). All statistical analyses were performed using the meta package of R software (35, 36).

## 3 Results

**Figure 1:**
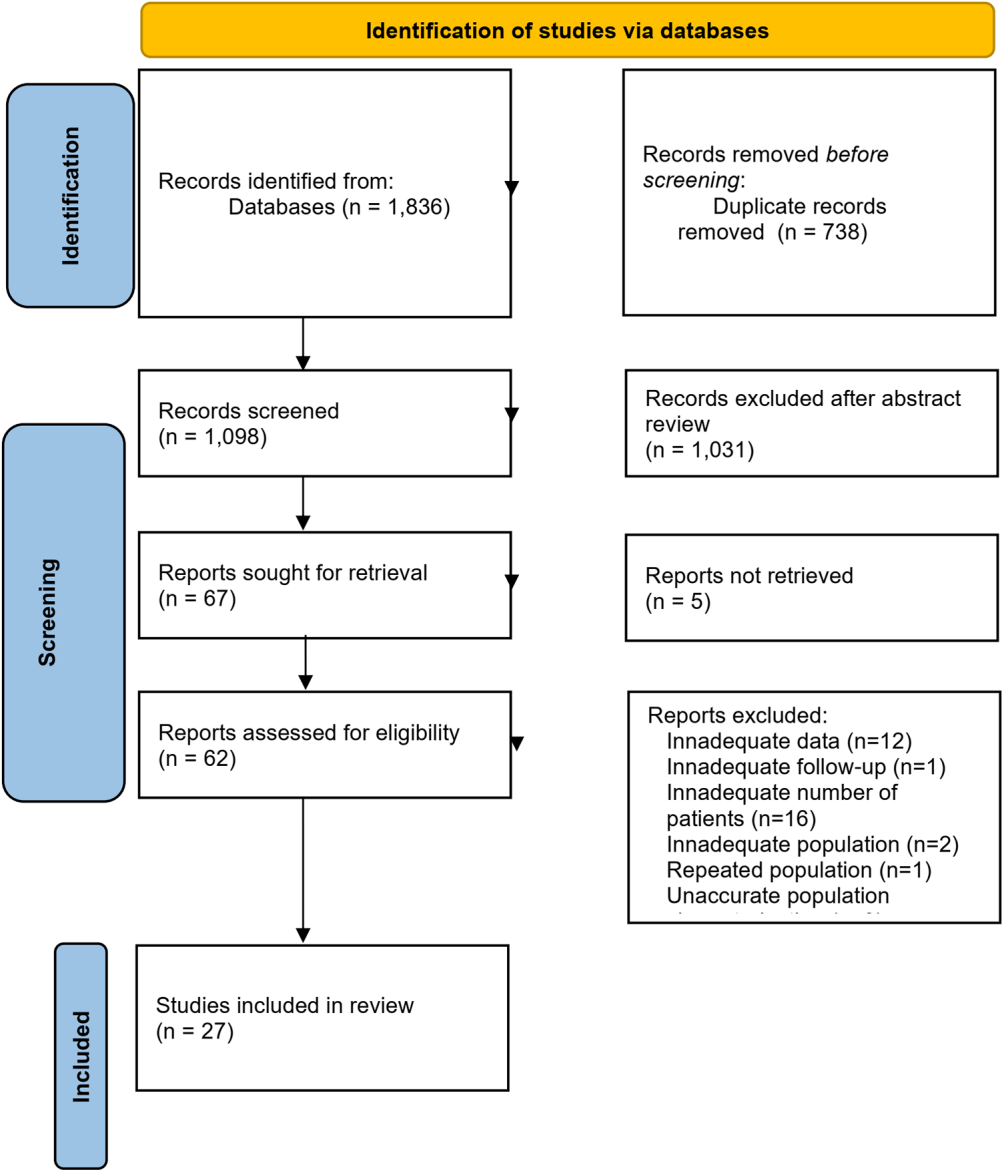
Flowchart for the study selection process. From: (37).

In total, 1,836 publications were identified through our search of MEDLINE/PubMed (944 records) and Web of Science (892 records) databases. After removing the duplicates, 1,098 records remained. Following the title and abstract screening, we selected 67 articles for full-text review. After excluding articles that did not meet the inclusion criteria, we ended up with 27 primary studies (see Figure 1 for the PRISMA 2020 flow diagram and Table 4 for a summary table of the included studies) (8–34).

**Table 4:** General characteristics of the included studies.

	Number of patients	Key inclusion criteria	Key exclusion criteria	Procedure	Outcomes	Type of study
Campos et al. (8)	188	Receiving a Cryolife O’Brien prosthesis (stentless bioprosthesis) in the aortic position.	Sinotubular dilation; extensive calcification of the aortic root; unfavorable position of coronary ostia.	SAVR	AVA, AVAI, MAG, LVM, LVMI, EDLVD, ESLVD, LVEF, PWT, IVST	Single-center, prospective cohort
Gegenava et al. (9)	210	Severe AS.	Absence of non-contrast-enhanced CT of the aortic valve; lack of complete echocardiographic follow-up.	TAVI	AVA, MAG, LVMI, EDLVD, ESLVD, LVEF	Single-center, RCT
Ngo et al. (10)	113	Symptomatic severe AS or left ventricular hypertrophy, decreased LVEF, or atrial fibrillation; >70 years.	Isolated AR; other significant valve diseases requiring intervention; CAD requiring revascularization; previous open-heart surgery; AMI or PCI within the last year; stroke or TIA within the last 30 days; renal insufficiency requiring hemodialysis; pulmonary insufficiency; active infectious disease requiring antibiotics; emergency intervention; unstable pre-interventional condition requiring inotropic support or mechanical heart assistance.	TAVI	AVA, AVAI, LVM, LVMI, EDLVD, ESLVD, EDLVV, ESLVV, LVEF, PWT, IVST	Single-center, retrospective cohort
Gelsomino et al. (11)	119	AVR with a CLOB stentless valve.	Contraindications for stentless valve implantation: extensive calcification of the sinus aortic wall and root; annulus diameter more than 30 mm that precluded the use of a 29-mm valve; extremely thin aortic wall.	SAVR	AVA, AVAI, MAG, LVMI, EDLVD, ESLVD, LVEF, IVST	Single-center, prospective cohort
Vizzardi et al. (12)	135	Symptomatic critical AS, with or without AR; age ≥75 years; logistic European System for Cardiac Operative Risk Evaluation score ≥15%; age ≥65 years and one or more of the following: cirrhosis (Child class A or B), pulmonary insufficiency, pulmonary hypertension, previous coronary artery bypass graft surgery or valvular surgery, porcelain aorta, recurrent pulmonary emboli, right ventricular insufficiency, contraindication to open-chest surgery, cachexia (BMI ≤18 kg/m^2^).	AMI in the preceding 30 days; PCI <15 days before implantation or scheduled during or within 30 days after TAVI; uncontrolled atrial fibrillation; history of AVR; stroke within the previous month; symptomatic carotid or vertebral artery disease (>70% stenosis); abdominal aortic aneurysm; bleeding diathesis or coagulopathy; eGFR<20 ml/m; life expectancy <1 year.	TAVI	AVAI, MAG, LVM, LVMI, EDLVV, ESLVV, LVEF, PWT, IVST	Single-center, prospective cohort
Pibarot et al. (13)	948	Severe AS and NYHA Functional Class ≥2, limited exercise capacity, abnormal BP response, or arrhythmia; severe AS with LVEF <50%; Heart Team agreement of a low operative mortality risk and an STS <4.	Anatomical contraindications for TAVI; AMI ≤1 month; unicuspid, bicuspid, or non-calcified aortic valve; severe AR; severe MR; ≥ moderate MS; pre-existing mechanical or bioprosthetic valve in any position; complex CAD; unprotected left main coronary artery; syntax score >32 (in the absence of prior revascularization); symptomatic carotid or vertebral artery disease or successful treatment of carotid stenosis within 30,days of randomization; leukopenia (WBC <3,000 cells/ml); anemia (Hgb <9 g/dl); thrombocytopenia (Plt <50,000 cells/ml); history of bleeding diathesis, coagulopathy, or hypercoagulable state; hemodynamic or respiratory instability requiring inotropic support, mechanical ventilation or mechanical heart assistance within 30 days of randomization; HCM with obstruction; LVEF <30%; intracardiac mass, thrombus or vegetation; stroke or TIA within 90 days of randomization; renal insufficiency (eGFR <30 ml/min) and/or renal replacement therapy at the time of screening; active bacterial endocarditis within 180 days of randomization; severe lung disease or currently on home oxygen; severe pulmonary hypertension; cirrhosis or any active liver disease; significant frailty as determined by the Heart Team; BMI >50 kg/m^2^; estimated life expectancy <24 months.	SAVR TAVI	AVA, AVAI, MAG, LVMI, EDLVD, ESLVD, LVEF	Multi-center, RCT
Izumi et al. (14)	269	AVR for chronic aortic valve disease.	Concomitant mitral valve replacement; acute AR due to aortic dissection or infective endocarditis.	SAVR	LVMI, EDLVD, ESLVD, LVEF	Multi-center, retrospective registry
Harrington et al. (15)	156	Severe AS submitted to TAVI; echocardiogram at least 1 day prior to TAVI and up to 1-year after the procedure.	NR	TAVI	MAG, LVM, LVMI, EDLVD, ESLVD, EDLVV, ESLVV, LVEF, PWT, IVST	Single-center, retrospective cohort
Merdler et al. (16)	224	TAVI for symptomatic severe AS with intermediate or high-risk for surgery.	AR or MR; patients with missing data.	TAVI	EDLVD, ESLVD, LVEF	Single-center, retrospective cohort
Martinovic et al. (17)	189	AVR with the CryoLife-O’Brien model 300 (stentless aortic porcine bioprosthesis).	Excessive calcification of the aortic root; aortic root aneurysm.	SAVR	AVA, MAG, LVMI	Single-center, prospective cohort
Al-Rashid et al. (18)	145	Severe symptomatic AS submitted to transfemoral TAVI; STS score ≥4% or considered excessive surgical risk due to comorbidities and other risk factors not reflected by the STS score.	Patients treated with a TAVI for the management of mitral valve pathology; pure non-calcific AR; previous or concomitant replacement of another valve; insufficient acoustic window preventing a complete echocardiographic study; hemodynamic instability.	TAVI	AVA, MAG, LVMI, EDLVV, ESLVV, LVEF	Single-center, prospective cohort
Thomson (19)	142	>59 years; predominant AS; AVR between December 1992 and February 1997 with either the CLOB or C-E xenografts or the ATS mechanical prosthesis.	Concomitant myomyectomy.	SAVR	AVA, LVM	Single-center, prospective cohort
Ewe et al. (20)	135	Symptomatic severe AS with high operative risk or the presence of contraindications to conventional aortic valve surgery.	Previous aortic or mitral prostheses; unsuccessful TAVI; echocardiographic follow-up <6 months.	TAVI	AVAI, MAG, LVMI, LVEF	Multi-center, prospective cohort
Douglas et al. (21)	143	Severe symptomatic AS.	AMI ≤1 month; unicuspid, bicuspid, or non-calcified aortic valve; mixed aortic valve disease; any therapeutic invasive cardiac procedure performed within 3 days of the index procedure; pre-existing prosthetic valve in any position; prosthetic ring; severe mitral annular calcification; severe MR; blood dyscrasias: leukopenia (WBC <3,000 mm^3^), acute anemia (Hgb <9 mg/dl), thrombocytopenia (platelet count <50,000 cells/mm^3^), history of bleeding diathesis or coagulopathy; untreated CAD requiring revascularization; hemodynamic instability requiring inotropic therapy or mechanical hemodynamic support devices; need for emergency surgery; HCM; LVEF <20%; intracardiac mass, thrombus or vegetation; active peptic ulcer or upper GI bleeding within the prior 3 months; recent stroke or TIA; renal insufficiency (creatinine >3.0 mg/dl) and/or ESRD requiring chronic dialysis; life expectancy <12 months; active bacterial endocarditis or other active infections; bulky calcified aortic valve leaflets in close proximity to coronary ostia; anatomical contraindications for TAVI.	TAVI	AVA, AVAI, MAG, LVM, LVMI, EDLVD, LVEF, PWT, IVST	Multi-center, RCT
Ledwoch et al. (22)	118	Severe symptomatic AS.	1-year follow-up not reached; death; no transthoracic echocardiogram at follow-up.	TAVI	AVA, MAG, LVMI, EDLVD, ESLVD, LVEF, PWT, IVST	Single-center, prospective cohort
Al-Hijji et al. (23)	101	Balloon-expandable TAVI using a Sapien valve.	Self-expanding CoreValve patients excluded from the transfemoral arm.	TAVI	AVAI, MAG, LVMI, EDLVD, LVEF	Single-center, retrospective cohort
Weber et al. (24)	149	Moderate to severe AS.	Relevant disease of other valves; AMI (<30 days); peripheral artery disease (>Fontaine stage IIb); LVEF < 30%; thrombotic embolism (<6 months); autoimmune disorders; renal failure (liable to dialysis); previous cardiac surgery; AR and dilatation of the ascending aorta receiving additional aortic surgery; TAVI or no surgical AVR decision.	SAVR	AVA, AVAI, MAG, LVM, LVMI, EDLVD, LVEF, PWT, IVST	Single-center, prospective cohort
Fuster et al. (25)	204	Pure or predominant AS.	Significant AR; coronary artery bypass surgery and other valve or aortic surgical procedures; emergent operations; infectious endocarditis; absence of preoperative echocardiography; previous AVR.	SAVR	AVA, MAG, LVMI, EDLVD, LVEF, PWT, IVST	Single-center, retrospective cohort
Theron et al. (26)	149	Severe AS.	NA	SAVR	AVA, AVAI, MAG, LVM, EDLVV, ESLVV, LVEF, IVST	Single-center, prospective cohort
Chau et al. (27)	1434	Symptomatic severe AS.	Exclusion criteria of the PARTNER 1A, 2A, and S3 trials and registries; missing LVMi data at 1 year.	TAVI	AVAI, MAG, LVMI, EDLVD, ESLVD, LVEF, PWT, IVST	Multi-center, RCT, registries
Ochiai et al. (28)	560	Symptomatic severe AS.	Death within 6 months of the procedure; lack of data from the 6-month follow-up; only one prescription of ACE inhibitors or ARBs during the follow-up (cross-over).	TAVI	AVA, AVAI, MAG, LVMI, EDLVV, ESLVV, LVEF	Multi-center, prospective cohort
Little et al. (29)	742	Symptomatic severe AS with increased risk for SAVR.	AMI ≤ 30 days; PCI or peripheral intervention performed within 30 days prior to the procedure; blood dyscrasias; CAD requiring revascularization; cardiogenic shock; need for emergency surgery; LVEF <20%; recent cerebrovascular accident or TIA; ESRD requiring chronic dialysis; eGFR <20 ml/min; GI bleeding within the last 3 months; ongoing sepsis; life expectancy <1 year; symptomatic carotid or vertebral artery disease; known hypersensitivity or contraindication to some drugs; participation in other trials; native aortic annulus size >29/<18 mm; pre-existing prosthetic valve in any position; bicuspid or unicuspid valve; mixed aortic valve disease; moderate to severe MR or tricuspid regurgitation; moderate to severe MS; obstructive HCM; intracardiac mass, thrombus or vegetation; severe basal septal hypertrophy with outflow gradient; specific anatomical contraindications.	TAVI and SAVR	AVA, AVAI, MAG, LVM, LVMI, EDLVV, ESLVV, EDLVD, ESLVD, LVEF, PWT, IVST	Multi-center, RCT
Ninomiya et al. (30)	100	Severe AS.	Death within 3 months after TAVI of causes unrelated to the procedure; absence of the 3-month follow-up echocardiogram.	TAVI	AVAI, MAG, LVMI, EDLVD, ESLVD, EDLVVI, ESLVVI, LVEF, PWT, IVST	Single-center, prospective cohort
Iliopoulos et al. (31)	121	AS or AR or mixed lesions (6.3% of patients with severe AR; 39.8% with mixed pathology).	Annuloaortic ectasia.	SAVR	MAG, EDLVD, ESLVD, IVST	Single-center, prospective cohort
Beholz et al. (32)	194	SAVR of the affected native or prosthetic aortic valve (6% for AR; 22% for AS + AR).	eGFR <20 ml/min; disorder of calcium metabolism; collagen autoimmune disease; active endocarditis; bicuspid aortic valve; coronary ostia and sinuses of Valsalva asymmetry; participation in other studies; additional valve replacement; previously implanted prosthetic valve other than aortic, which is to be replaced; intravenous drug abuse; HIV-positive; life expectancy <3 years; HCM.	SAVR	AVA, AVAI, MAG, LVM, EDLVD, ESLVD, LVEF, PWT, IVST	Multi-center, prospective cohort
Fischlein et al. (33)	137	AS or AS + AR (34.3%); age >65 years.	Participation in other studies; previously implanted Perceval prosthesis requiring replacement; previous implantation of valve prostheses or annuloplasty ring not being replaced by the study valve; need of simultaneous cardiac procedures (except septal myectomy, coronary artery bypass grafting, or both); need for multiple valve replacement or repair that would be replaced with a non-Perceval valve or repaired; ascending aorta dissection or aneurysm; non-elective intervention; active endocarditis or myocarditis; bicuspid aortic valve; aortic root enlargement; AMI within 90 days before the planned surgery; hypersensitivity to nickel alloys; life expectancy <1 year; unacceptably high surgical risk; renal dialysis; chronic renal failure with hyperparathyroidism; acute preoperative neurological deficit; AMI or cardiac event that has not returned to baseline or stabilized at least 30 days before the valve surgery.	SAVR	AVA, MAG, LVMI	Multi-center, prospective cohort
Medvedofsky et al. (34)	123	Severe symptomatic AS.	Presence of a pacemaker; poor-quality image; atrial fibrillation.	TAVI	EDLVVI, ESLVVI, LVEF	Multi-center, prospective cohort

ACE inhibitors, angiotensin-converting enzyme inhibitors; AR, aortic regurgitation; ARBs, angiotensin receptor blockers; AMI, acute myocardial infarction; AS, aortic stenosis; AVA, aortic valve area; AVAI, aortic valve area index; AVR, aortic valve replacement; BMI, body mass index; BP, blood pressure; CAD, coronary artery disease; CT, computed tomography; EDLVD, end-diastolic left ventricular diameter; EDLVVI, end-diastolic left ventricular volume index; EDLVV, end-diastolic left ventricular volume; eGFR, estimated glomerular filtration rate; ESLVD, end-systolic left ventricular diameter; ESLVVI, end-systolic left ventricular volume index; ESLVV, end-systolic left ventricular volume; ESRD, end stage renal disease; GI, gastrointestinal; HCM, hypertrophic cardiomyopathy; HIV, human immunodeficiency virus; Hgb, hemoglobin; IVST, interventricular septal thickness; LVEF, left ventricle ejection fraction; LVM, left ventricular mass; LVMI, left ventricular mass index; MAG, mean aortic gradient; MR, mitral regurgitation; MS, mitral stenosis; NR, not reported; NYHA, New York Heart Association (NYHA) Classification; PCI, percutaneous coronary intervention; Plt, platelet; PWT, posterior wall thickness; RCT, randomized controlled trial; SAVR, surgical aortic valve replacement; STS Score, Society of Thoracic Surgery Score; TIA, transient ischemic attack; TAVI, transcatheter aortic valve implantation; WBC, white blood cell.

Since some studies contained more than one distinct population, the search yielded 39 independent patient cohorts. The studies were published between 1998 and 2020, assessing 11,751 patients who completed echocardiographic assessment before and at least 1 month post-AVR.

### 3.1 Effective aortic valve area and mean aortic gradient

While this work is related to left ventricular remodeling after AVR, we chose to start by reporting measures related to AVR, such as aortic valve area and gradient. This ensures that the studies assessed comparable conditions and demonstrated similar improvements after valve obstruction is resolved. By doing so, we aimed to establish a consistent baseline for analyzing left ventricular remodeling parameters.

**Figure 2:**
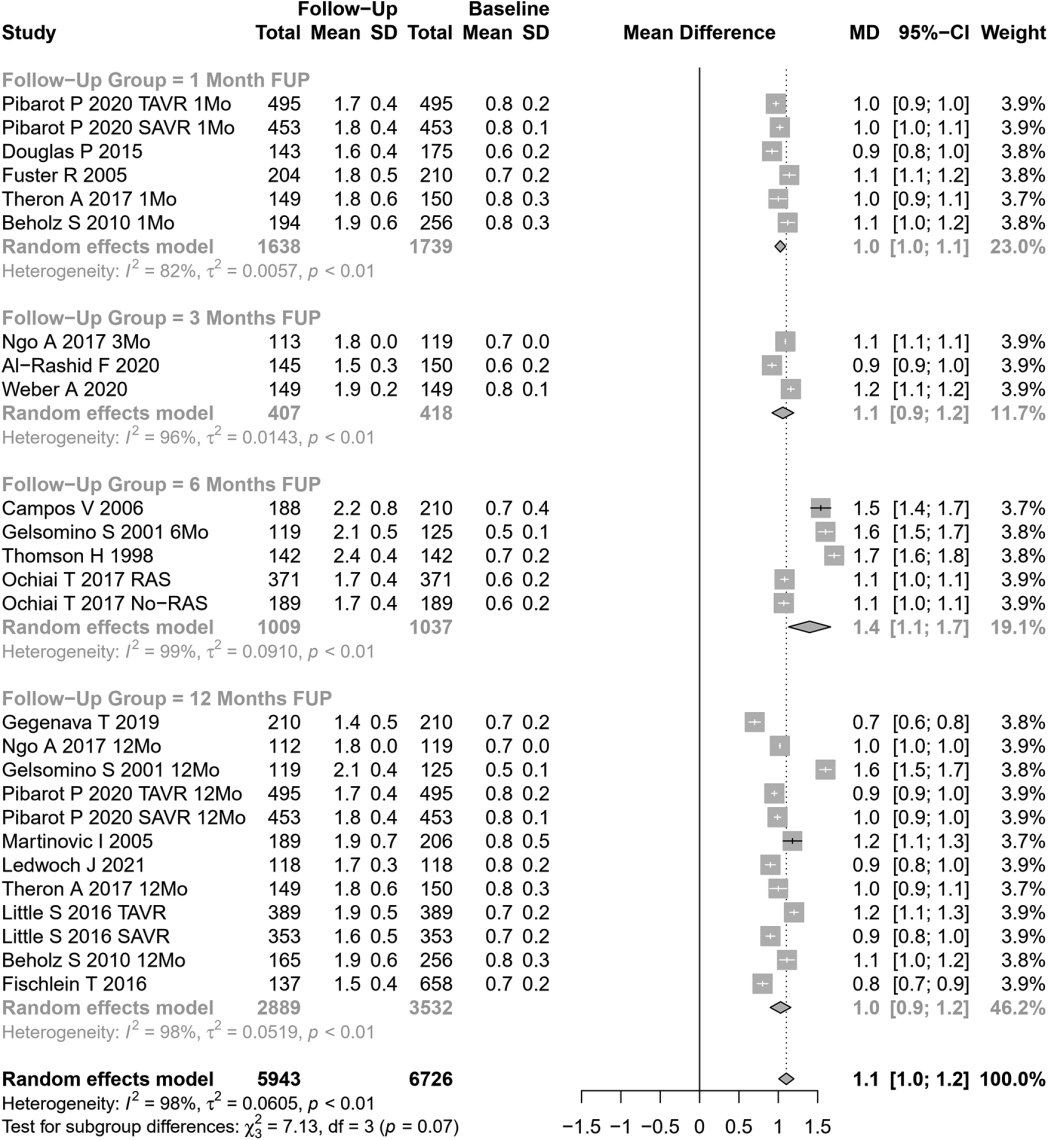
SMD post-AVR vs. pre-AVR for the aortic valve area.

Our meta-analytical results indicate that, after AVR, there was an increase in the effective aortic valve area and a decrease in the mean aortic gradient. Based on 26 cohorts (n=6,726 at baseline, Figure 2), the pooled SMD for effective aortic valve area was 1.10 cm^2^ (95% CI: 1.01–1.20, p<0.0001, $I^{2}=98\%$, Cochran’s Q p-value <0.0001), corresponding to a significant increase after AVR, albeit with substantial heterogeneity.Univariate meta-regression identified publication year, age, hypertension, NYHA class III or IV, DM, type of AVR, and EF >50% as potential moderators of heterogeneity (see Supplementary Table S1 for subgroup and heterogeneity analysis and Supplementary Table S2 for meta-regression).

In studies assessing SAVR (15 cohorts), AVA increased by 1.19 cm^2^ (95% CI: 1.05– 1.33), while in TAVI patients (11 cohorts), AVA increased by 0.99 cm^2^ (95% CI: 0.91– 1.06). The results were significantly different between SAVR and TAVI patients (p=0.01). No significant differences were observed when our results were stratified according to the follow-up period (Figure 2).

**Figure 3:**
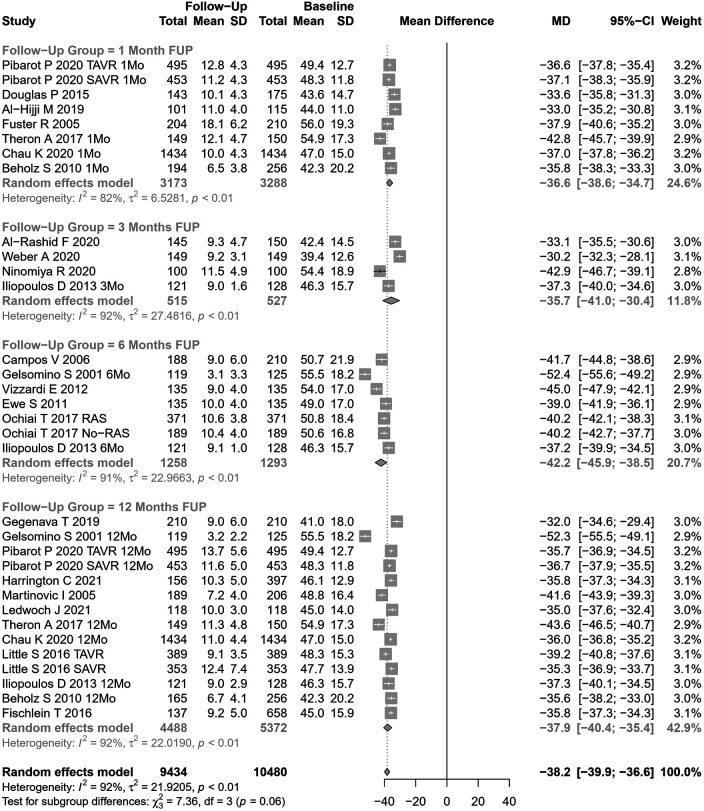
SMD post-SAVR vs. pre-SAVR for MAG.

The mean aortic gradient was assessed in 33 cohorts (n=10,480 patients at baseline, Figure 3). The pooled SMD for mean aortic gradient was −38.23 mmHg (95% CI: −39.88 to −36.58 mmHg, p<0.0001, $I^{2}=92\%$, Cochran’s Q p-value <0.0001), indicating a significant decrease after AVR, but with substantial heterogeneity. Univariate meta-regression identified publication year and coronary artery disease as potential moderators of heterogeneity (see Supplementary Table S1 for subgroup and heterogeneity analysis and Supplementary Table S2 for meta-regression). Subgroup analyses showed a trend for differences according to follow-up periods (p=0.06; Figure 3) but not according to the type of AVR (p=0.16).

### 3.2 Parameters on left ventricular reverse remodeling

#### 3.2.1 Left ventricular mass

**Figure 4:**
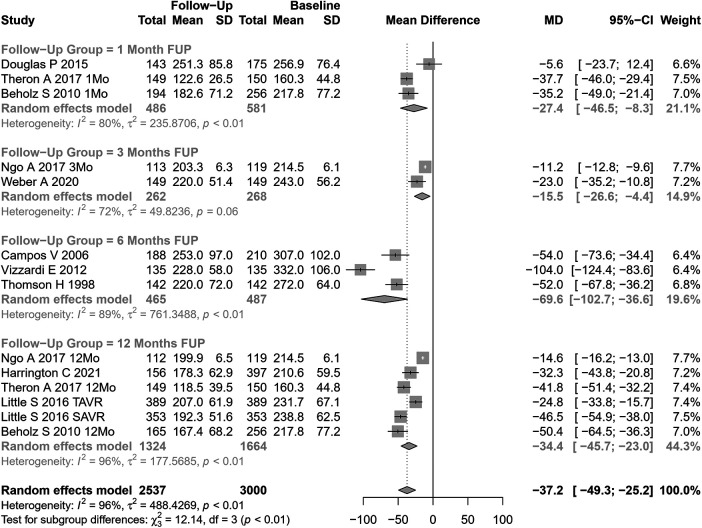
SMD post-AVR vs. pre-AVR for LVM.

LVM change after AVR was analyzed in 14 cohorts (Figure 4). The pooled SMD for LVM was −37.24 g (95% CI: −49.31 to −25.18, p<0.0001; $I^{2}=96\%$, Cochran’s Q p-value <0.0001), indicating a significant decrease after AVR, albeit with substantial heterogeneity.

Performing subgroup analysis according to follow-up periods, significant differences were observed (p=0.007). However, the values involved were relatively small (and may represent different samples evaluated at various time points and not a cohort evaluated prospectively through time): LVM reduction of 27 g at 1 month, 16 g at 3 months, 70 g at 6 months, and 34 g at  12 months. Performing subgroup analysis according to the type of AVR, no significant differences were observed (p=0.49).

Univariate meta-regression identified publication year and DM as potential moderators of heterogeneity (see Supplementary Table S1 for subgroup and heterogeneity analysis and Supplementary Table S2 for meta-regression).

#### 3.2.2 Left ventricular ejection fraction

**Figure 5:**
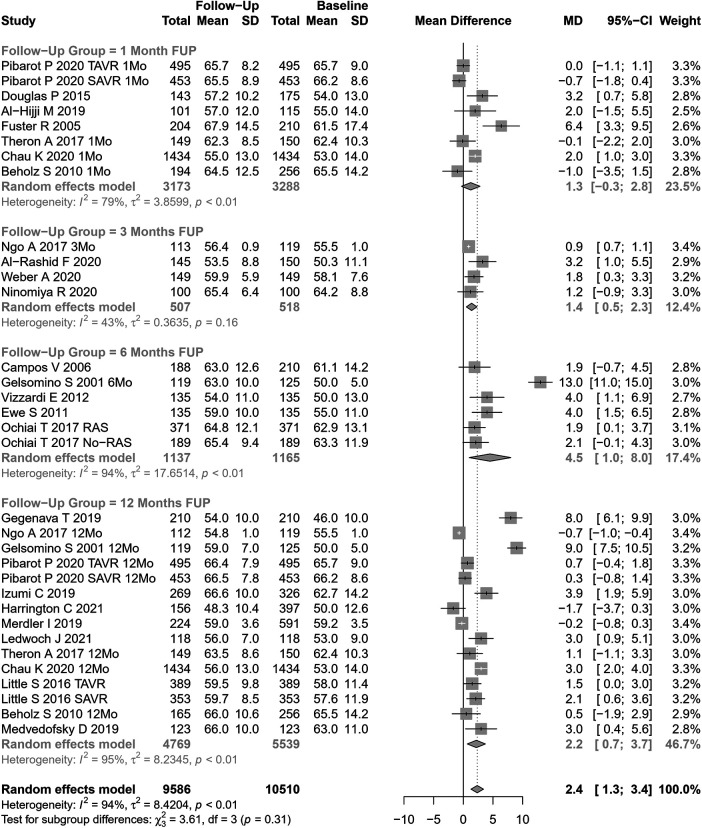
SMD post-AVR vs. pre-AVR for LVEF.

LVEF change after AVR was assessed in 33 cohorts (n=10,510 participants at baseline, Figure 5). The pooled SMD for LVEF was 2.35% (95% CI: 1.31%–3.40%, p<0.0001; $I^{2}=94.1\%$, Cochran’s Q p-value <0.0001), indicating a significant increase after AVR, although with substantial heterogeneity. Performing subgroup analysis according to follow-up periods or the type of AVR, no significant differences were observed (p=0.31 and p=0.42, respectively).

Univariate meta-regression identified publication year and NYHA classification III or IV as potential moderators of heterogeneity (Supplementary Table S1 for subgroup and heterogeneity analysis and Supplementary Table S2 for meta-regression).

#### 3.2.3 End-diastolic left ventricular diameter and volume

**Figure 6:**
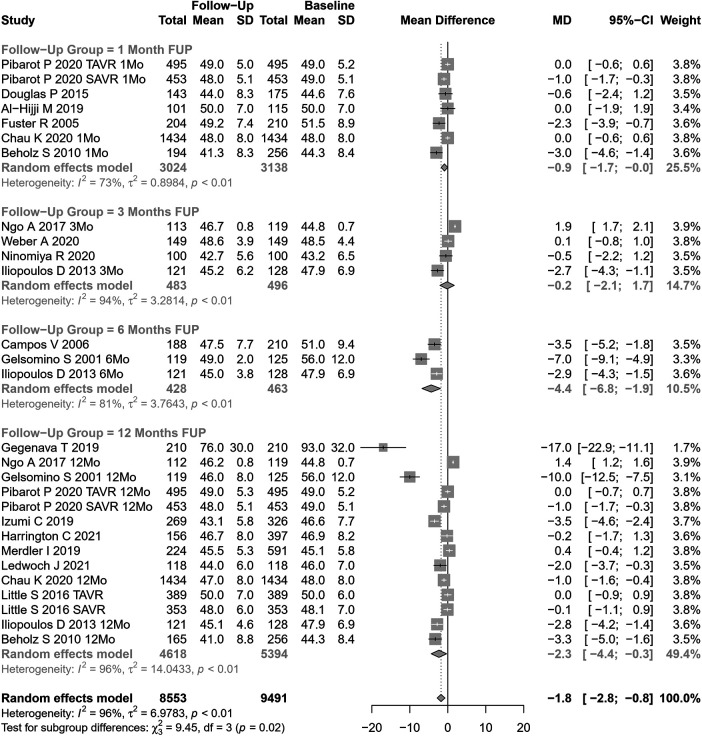
SMD post-AVR vs. pre-AVR for EDLVD.

EDLVD change after AVR was assessed in 28 cohorts (n=9,491 participants at baseline, Figure 6). The pooled SMD for EDLVD was −1.78 mm (95% CI: −2.80 to −0.76, p=0.0006; $I^{2}=96\%$, Cochran’s Q p-value <0.0001), indicating a significant decrease after AVR, although with substantial heterogeneity.

Stratifying our results according to follow-up periods, significant differences were observed (p=0.02). However, the values involved were relatively small (and may represent different samples evaluated at various time points, rather than a cohort evaluated prospectively through time): EDLVD decreased by 0.88 mm at 1 month, 0.18 mm at 3 months, 6.77 mm at 6 months, and 2.33 mm at 12 months.

Significant differences were also observed in performing subgroup analysis according to the type of AVR (p=0.0002). In studies assessing SAVR (14 cohorts), EDLVD decreased by 2.92 mm (95% CI: −4.21 to −1.63) vs 0.16 mm in TAVI patients (14 cohorts; 95% CI: −0.87 to −0.55). Univariable meta-regression identified publication year, age, and coronary artery disease as potential moderators of heterogeneity (see Supplementary Table S1 for subgroup and heterogeneity analysis and Supplementary Table S2 for meta-regression).

**Figure 7:**
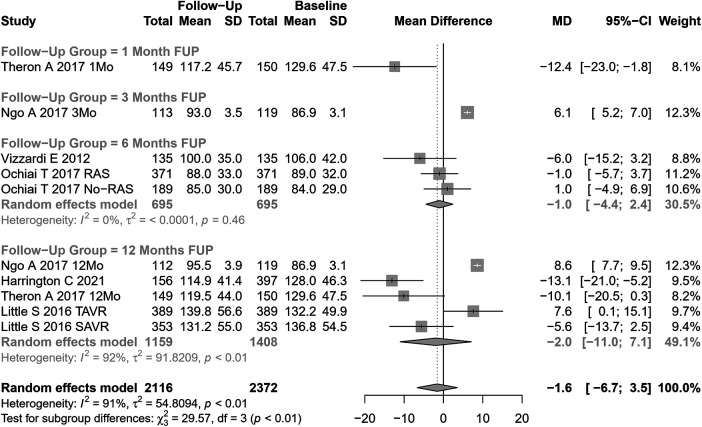
SMD post-SAVR vs. pre-SAVR for EDLVV.

EDLVV change after AVR was assessed in 10 cohorts (n=2,116 participants at baseline, Figure 7). The pooled SMD for EDLVV was −1.6 ml (95% CI: −6.68 to 3.51, p=0.54; $I^{2}=91\%$, Cochran’s Q p-value <0.001), indicating a non-significant decrease after AVR.

Univariate meta-regression identified the type of AVR, coronary artery disease, and hypertension as potential moderators of heterogeneity (see Supplementary Table S1 for subgroup and heterogeneity analysis and Supplementary Table S2 for meta-regression).

## 4 Discussion

In this study, we assessed the echocardiographic parameters of the unloaded LV after AVR. Notably, LV reverse remodeling was evident at the earliest time point evaluated (1 month after AVR). Several of the evaluated parameters were consistent with reverse remodeling, namely, the significant reduction observed in LVM and EDLVD, and LVEF improvement. A trend for EDLVV reduction was also observed. Our results are consistent with those from Mehdipoor et al. (38), who reported indexed LVM reduction and increased LVEF within 6–15 months after TAVI on 10 primary studies involving 305 patients.

Patient follow-up after AVR typically focusses on monitoring valve hemodynamics over time, specifically the evolution of the effective aortic valve area, gradient, and left ventricular function. Reverse left ventricular remodeling is not commonly assessed in routine clinical practice post-AVR. This is partly due to the lack of established norms for what constitutes “normal” left ventricular remodeling after AVR. This study aimed to establish a framework for the expected changes in certain parameters following AVR.

Finally, it is important to note that, despite its infrequent use, the extent of left ventricular remodeling has significant prognostic implications post-AVR. Patients who do not exhibit improvements in LVEF and reductions in left ventricular mass and dimensions after AVR are at a higher risk for increased cardiovascular events (14, 39). In our opinion, further attention should be paid to the predictors of inadequate left ventricular remodeling after AVR, as this may aid in defining other criteria for AVR other than the severity of obstruction and left ventricular function.

### 4.1 Strengths and limitations

To our knowledge, this is the most extensive systematic review and meta-analysis conducted to assess the reverse LV remodeling profile in patients who underwent AVR. We excluded studies without a predefined follow-up period to obtain the most robust results possible. We performed meta-regression and subgroup analyses to explore sources of heterogeneity, identifying several variables in this context. To minimize publication and information bias, we searched different electronic bibliographic databases without applying exclusion criteria based on the date or language of publication and contacted authors whenever relevant information was missing.

Limitations of this meta-analysis are related to three main factors: the inherent source of variability regarding to measurements performed by echocardiography, the incomplete characterization of patients in some of the included studies, and the significant heterogeneity observed in our results.

First, a significant source of variability may be related to the fact that primary studies used TTE as the imaging LV assessment method, which is affected by inter-observer and intra-observer variability that can be a source of heterogeneity. For example, the non-significant reduction in LV volume compared to a significant reduction in LV diameter likely reflects the higher variability in echocardiographic measurements of three-dimensional parameters like LV volume, which tend to have a higher standard deviation compared to two-dimensional measurements like LV diameter. This variability could obscure significant findings. An analysis based on studies using CMR to evaluate LV could possibly reduce the heterogeneity across studies. However, it would be an undoubtedly less clinically useful analysis (40–42). Finally, another possible source of heterogeneity is the presence of prosthesis–patient mismatch (PPM), which could influence the results by leading to worse hemodynamic function and LV reverse remodeling. Our study did not analyze PPM because it was not reported in most studies.

Second, other non-evaluated factors may influence the extent of left ventricular remodeling after AVR. In this work, we showed that LV reverse remodeling may differ according to several patient characteristics, namely, age, hypertension, diabetes, coronary heart disease, and NYHA classification. However, the data available for analysis were sparse on information regarding the severity and duration of aortic stenosis, pre-existing LV remodeling, the presence of atrial fibrillation, associated valvular heart diseases, diastolic function, and patient–prosthesis mismatch that may also contribute to the extent of reverse remodeling. Furthermore, by using a summary or aggregate data from study publications, our meta-analysis may fail to identify patient characteristics that might be significant predictors of adequate LV remodeling. For example, previous works have shown that women have a more favorable LV remodeling after AVR than men (43). However, the available aggregate data were insufficient to characterize the impact of gender on LV reverse remodeling after AVR.

Finally, significant heterogeneity among studies was observed. Even though meta-regression and subgroup analysis were performed to identify possible variables that differed between studies and could explain the differences between primary studies, it must be noted that the included studies were mainly observational studies and included patients based on convenient criteria (i.e., patients who underwent AVR at a given institution), which added significant heterogeneity that cannot be controlled using regression techniques.

## 5 Conclusion

This is the most extensive systematic review and meta-analysis assessing reverse LV remodeling after AVR. Echocardiography demonstrates reverse LV remodeling as soon as 1 month after AVR, with reductions in MAG, LVM, and EDLVD, and improvement in AVA and LVEF.

## Data Availability

The original contributions presented in the study are included in the article/Supplementary Material, further inquiries can be directed to the corresponding author.
