# Corrigendum: Reverse left ventricular remodeling after aortic valve replacement for aortic stenosis: a systematic review and meta-analysis

**DOI:** 10.3389/fcvm.2024.1471225

**Published:** 2024-08-13

**Authors:** F. Sousa Nunes, C. Amaral Marques, A. Isabel Pinho, B. Sousa-Pinto, A. Beco, J. Ricardo Silva, F. Saraiva, F. Macedo, A. Leite-Moreira, C. Sousa

**Affiliations:** ^1^ Cardiovascular R&D Centre—UnIC@RISE, Department of Surgery and Physiology, Faculty of Medicine of the University of Porto, Porto, Portugal; ^2^ Department of Cardiology, Local Health Unit of Gaia and Espinho, Vila Nova de Gaia, Portugal; ^3^ Department of Cardiology, Local Health Unit of Sao Joao, Porto, Portugal; ^4^ MEDCIDS—Department of Community Medicine, Information and Health Decision Sciences, Faculty of Medicine, University of Porto, Porto, Portugal; ^5^ CINTESIS@RISE—Health Research Network, MEDCIDS, Faculty of Medicine, University of Porto, Porto, Portugal

**Keywords:** aortic stenosis, transcatheter aortic valve implantation (TAVI), surgical aortic valve replacement (SAVR), reverse left ventricle remodeling, echocardiography

A Corrigendum on Reverse left ventricular remodeling after aortic valve replacement for aortic stenosis: a systematic review and meta-analysis Sousa Nunes F, Amaral Marques C, Isabel Pinho A, Sousa-Pinto B, Beco A, Ricardo Silva J, Saraiva F, Macedo F, Leite-Moreira A and Sousa C (2024). Front. Cardiovasc. Med. 11:1407566. doi: 10.3389/fcvm.2024.1407566

In the published article, there was an error in the Funding statement. In the original article it is written “The author(s) declare that no financial support was received for the research, authorship, and/or publication of this article.” The correct Funding statement appears below.

