# Supplementary material for: Reverse left ventricular remodeling after aortic valve replacement for aortic stenosis: a systematic review and meta-analysis
Source: Front Cardiovasc Med. 2024 Jul 4;11:1407566. doi: 10.3389/fcvm.2024.1407566 (PMC11254856; doi:10.3389/fcvm.2024.1407566)
Supplement: Supplementary file 1 [file Datasheet1.docx]

**Table. S1** Meta-analytical results and results of the subgroup analysis as moderators of heterogeneity

|  | n cohorts | Subgroup analysis | | |
| --- | --- | --- | --- | --- |
|  |  | SMD (95% CI) | Heterogeneity – I^2^ | p-value subgroup |
| Aortic Valve Area |  |  |  |  |
| Follow-Up Time |  |  |  | 0.07 |
| 1 Mo | 6 | 1.03 (0.96-1.09) | 81.7% |  |
| 3 Mo | 3 | 1.06 (0.92-1.20) | 95.7% |  |
| 6 Mo | 5 | 1.40 (1.13-1.67) | 98.7% |  |
| 12 Mo | 12 | 1.03 (0.90-1.16) | 97.6% |  |
| Ejection Fraction |  |  |  | <0.0001 |
| < 50% | 1 | 0.70 (0.63-1.19) | 97.1% |  |
| > 50% | 22 | 1.10 (1.02-1.19) | - |  |
| Type of Procedure |  |  |  | 0.01 |
| TAVI | 11 | 0.99 (0.91-1.06) | 98.2% |  |
| SAVR | 15 | 1.19 (1.05-1.33) | 97.1% |  |
| Mean Aortic Gradient |  |  |  |  |
| Follow-Up Time |  |  |  | 0.06 |
| 1 Mo | 8 | -36.6 (-38.6 - -34.7) | 81.7% |  |
| 3 Mo | 4 | -35.7 (-41.0 - -30.4) | 92.5% |  |
| 6 Mo | 7 | -42.2 (-45.9 - -38.5) | 90.6% |  |
| 12 Mo | 14 | -37.9 (-40.4 - -35.4) | 92.2% |  |
| Ejection Fraction |  |  |  | 0.0002 |
| < 50% | 1 | -32.0 (-34.6 - -29.4) | - |  |
| > 50% | 20 | -38.7 (-41.1 - -36.2) | 94.0% |  |
| Type of Procedure |  |  |  | 0.16 |
| TAVI | 16 | -37.0 (-38.7 - -35.3) | 87.7% |  |
| SAVR | 17 | -39.4 (-42.1 - -36.6) | 94.2% |  |
| Left Ventricular Mass |  |  |  |  |
| Follow-Up Time |  |  |  | 0.007 |
| 1 Mo | 3 | -27.4 (-46.5 - -8.3) | 80.3% |  |
| 3 Mo | 2 | -15.5 (-26.6 - -4.4) | 71.6% |  |
| 6 Mo | 3 | -69.6 (-102.7 - -36.6) | 88.8% |  |
| 12 Mo | 6 | -34.4 (-45.7 - -23.0) | 95.5% |  |
| Ejection Fraction |  |  |  | -- |
| < 50% | 0 |  |  |  |
| > 50% | 13 | -36.2 (-49.0 - -23.4) | 95.6% |  |
| Type of Procedure |  |  |  | 0.48 |
| TAVI | 6 | -31.4 (-59.4 - -3.3) | 95.1% |  |
| SAVR | 8 | -41.6 (-48.3 - -35.0) | 57.6% |  |

(continued)

|  | n cohorts | Subgroup analysis | | |
| --- | --- | --- | --- | --- |
|  |  | SMD (95% CI) | Heterogeneity – I^2^ | p-value subgroup |
| Left Ventricular Ejection Fraction |  |  |  |  |
| Follow-Up Time |  |  |  | 0.31 |
| 1 Mo | 8 | 1.3 (-0.3 – 2.8) | 79.2% |  |
| 3 Mo | 4 | 1.4 (0.5 – 2.3) | 42.7% |  |
| 6 Mo | 6 | 4.5 (1.0 – 8.0) | 94.2% |  |
| 12 Mo | 15 | 2.2 (0.7 – 3.7) | 95.5% |  |
| Ejection Fraction |  |  |  | < 0.0001 |
| < 50% | 1 | 8.0 (6.1 – 9.9) | - |  |
| > 50% | 32 | 2.2 (1.2 – 3.2) | 93.6% |  |
| Type of Procedure |  |  |  | 0.42 |
| TAVI | 20 | 1.9 (1.0 – 2.8) | 92.0% |  |
| SAVR | 13 | 2.9 (0.7 – 5.2) | 95.2% |  |
| LV End-Diastolic Diameter |  |  |  |  |
| Follow-Up Time |  |  |  | 0.02 |
| 1 Mo | 7 | -0.9 (-1.7 - -0.0) | 73.4% |  |
| 3 Mo | 4 | -0.2 (-2.1 – 1.7) | 94.0% |  |
| 6 Mo | 3 | -4.4 (-6.8 – -1.9) | 80.7% |  |
| 12 Mo | 14 | -2.3 (-4.4 – -0.3) | 96.1% |  |
| Ejection Fraction |  |  |  | <0.0001 |
| < 50% | 1 | -17.0 (-22.9 – -11.1) | -- |  |
| > 50% | 24 | -1.3 (-2.3 - -0.4) | 95.9% |  |
| Type of Procedure |  |  |  | 0.0002 |
| TAVI | 14 | -0.2 (-0.9 – 0.5) | 94.2% |  |
| SAVR | 14 | -2.9 (-4.2 – -1.6) | 89.7% |  |
| LV End-Diastolic Volume |  |  |  |  |
| Follow-Up Time |  |  |  | <0.0001 |
| 1 Mo | 1 | -12.4 (-23.0 – -1.8) | - |  |
| 3 Mo | 1 | 6.1 (5.2 – 6.9) | - |  |
| 6 Mo | 3 | -1.0 (-4.4 – 2.4) | 0.0% |  |
| 12 Mo | 5 | -2.0 (-11.0 – 7.1) | 92.2% |  |
| Ejection Fraction |  |  |  | -- |
| < 50% | - |  |  |  |
| > 50% | 10 | -1.6 (-6.7 – 3.5) | 90.6% |  |
| Type of Procedure |  |  |  | 0.01 |
| TAVI | 7 | 1.1 (-4.5 – 6.7) | 90.5% |  |
| SAVR | 3 | -8.7 (-14.1 - -3.2) | 0.0% |  |

Mo, Month; SAVR, Surgical Aortic Valve Replacement; SMD, Standardized Mean Difference; TAVI, Transcatheter Aortic Valve Implantation; 95% CI, 95% Confidence Interval

**Table. S2** Results of the meta-regression analysis

|  | Aortic Valve Area | | | | Mean Aortic Gradient | | | |
| --- | --- | --- | --- | --- | --- | --- | --- | --- |
|  | n cohorts | R^2^ | p-value |  | n cohorts | R^2^ | p-value |  |
| Follow-Up Time | 26 | 0% | 0.66 |  | 33 | 0% | 0.62 |  |
| LVEF | 23 | 10.9% | 0.06 |  | 28 | 2.0% | 0.22 |  |
| Type of Procedure | 26 | 14.2% | 0.02 |  | 33 | 2.4% | 0.18 |  |
| Publication Year | 26 | 70.5% | <0.0001 |  | 33 | 43.5% | <0.0001 |  |
| Age | 25 | 17.2% | 0.01 |  | 32 | 5.8% | 0.09 |  |
| Sex | 26 | 0% | 0.73 |  | 33 | 0% | 0.61 |  |
| BSA | 14 | 4.3% | 0.21 |  | 21 | 5.1% | 0.15 |  |
| HTN | 20 | 14.9% | 0.05 |  | 27 | 0% | 0.58 |  |
| Diabetes | 20 | 14.5% | 0.04 |  | 28 | 0% | 0.45 |  |
| NYHA class III or IV | 24 | 14.1% | 0.03 |  | 28 | 0.45% | 0.27 |  |
| CAD | 18 | 0% | 0.77 |  | 24 | 18.5% | 0.01 |  |

(continued)

|  | Left Ventricular Mass | | | | Left Ventricle Ejection Fraction | | | |
| --- | --- | --- | --- | --- | --- | --- | --- | --- |
|  | n cohorts | R^2^ | p-value |  | n cohorts | R^2^ | p-value |  |
| Follow-Up Time | 14 | 0% | 0.64 |  | 33 | 0% | 0.70 |  |
| LVEF at baseline | - |  |  |  | 33 | 9.3% | 0.05 |  |
| Type of Procedure | 14 | 1.13% | 0.34 |  | 33 | 0% | 0.40 |  |
| Publication Year | 14 | 16.2% | 0.08 |  | 33 | 41.1% | <0.0001 |  |
| Age | 13 | 0% | 0.68 |  | 32 | 3.5% | 0.14 |  |
| Sex | 14 | 0% | 0.78 |  | 33 | 1.3% | 0.28 |  |
| BSA | - |  |  |  | 20 | 1.7% | 0.24 |  |
| HTN | 10 | 0% | 0.59 |  | 27 | 8.7% | 0.05 |  |
| Diabetes | 10 | 66% | 0.0002 |  | 28 | 0% | 0.71 |  |
| NYHA class III or IV | 11 | 0% | 0.90 |  | 28 | 21.0% | 0.007 |  |
| CAD | 9 | 8.0% | 0.2 |  | 24 | 0.8% | 0.27 |  |

(continued)

|  | Left Ventricular End-Diastolic Diameter | | | | Left Ventricular End-Diastolic Volume | | | |
| --- | --- | --- | --- | --- | --- | --- | --- | --- |
|  | n cohorts | R^2^ | p-value |  | n cohorts | R^2^ | p-value |  |
| Follow-Up Time | 28 | 0% | 0.30 |  | 10 | 0% | 0.89 |  |
| LVEF at baseline | 25 | 34.6% | <0.0001 |  | - |  |  |  |
| Type of Procedure | 28 | 37.0% | 0.004 |  | 11 | 30.1% | 0.05 |  |
| Publication Year | 28 | 62.2% | <0.0001 |  | 10 | 0% | 0.58 |  |
| Age | 27 | 25.5% | 0.02 |  | 10 | 0% | 0.60 |  |
| Sex | 28 | 3.3% | 0.16 |  | 10 | 0% | 0.46 |  |
| BSA | 19 | 0% | 0.43 |  | 8 | 0% | 0.95 |  |
| HTN | 22 | 0% | 0.33 |  | 8 | 48.6% | 0.01 |  |
| Diabetes | 23 | 0% | 0.75 |  | 8 | 39.3% | 0.06 |  |
| NYHA class III or IV | 23 | 0% | 0.40 |  | 9 | 0% | 0.40 |  |
| CAD | 24 | 13.4% | 0.07 |  | 8 | 52.2% | 0.02 |  |

R^2^ is the amount of heterogeneity accounted for by the variable. The p-value for the test of moderators indicates whether the variable influences the effect size.

BSA, Body Surface Area; CAD, Coronary Artery Disease; HTN, Hypertension; LVEF, Left Ventricle Ejection Fraction; NYHA, New York Heart Association (NYHA) Classification
